# Clinical presentation and diagnosis of adult-onset leukoencephalopathy with axonal spheroids and pigmented glia: a literature analysis of case studies

**DOI:** 10.3389/fneur.2024.1320663

**Published:** 2024-03-11

**Authors:** Spyros Papapetropoulos, Jeffrey M. Gelfand, Takuya Konno, Takeshi Ikeuchi, Angela Pontius, Andreas Meier, Farid Foroutan, Zbigniew K. Wszolek

**Affiliations:** ^1^Vigil Neuroscience, Inc., Watertown, MA, United States; ^2^UCSF Medical Center, San Francisco, CA, United States; ^3^Brain Research Institute, Niigata University, Niigata, Japan; ^4^Department of Clinical Epidemiology and Biostatistics, McMaster University, Hamilton, ON, Canada; ^5^Mayo Clinic, Jacksonville, FL, United States

**Keywords:** adult-onset leukoencephalopathy with axonal spheroids and pigmented glia, ALSP, CSF1R-related leukoencephalopathy, diagnosis, misdiagnosis

## Abstract

**Introduction:**

Because adult-onset leukoencephalopathy with axonal spheroids and pigmented glia (ALSP) is a rare, rapidly progressive, debilitating, and ultimately fatal neurodegenerative disease, a rapid and accurate diagnosis is critical. This analysis examined the frequency of initial misdiagnosis of ALSP via comprehensive review of peer-reviewed published cases.

**Methods:**

Data were extracted from a MEDLINE search via PubMed (January 1, 1980, through March 22, 2022) from eligible published case reports/series for patients with an ALSP diagnosis that had been confirmed by testing for the colony-stimulating factor-1 receptor gene (*CSF1R*) mutation. Patient demographics, clinical symptoms, brain imaging, and initial diagnosis data were summarized descriptively. Categorical data for patient demographics, symptoms, and brain imaging were stratified by initial diagnosis category to test for differences in initial diagnosis based on each variable.

**Results:**

Data were extracted from a cohort of 291 patients with ALSP from 93 published case reports and case series. Mean (standard deviation) age of symptom onset was 43.2 (11.6) years. A family history of ALSP was observed in 59.1% of patients. Cognitive impairment (47.1%) and behavioral and psychiatric abnormalities (26.8%) were the most frequently reported initial symptoms. Of 291 total cases, an accurate initial diagnosis of ALSP was made in 72 cases (24.7%) and the most frequent initial misdiagnosis categories were frontotemporal dementia (28 [9.6%]) and multiple sclerosis (21 [7.2%]). Of the 219 cases (75.3%) that were initially mis- or undiagnosed, 206 cases (94.1%) were later confirmed as ALSP by immunohistology, imaging, and/or genetic testing; for the remaining 13 cases, no final diagnosis was reported. Initial diagnosis category varied based on age, family history, geographic region, mode of inheritance, and presenting symptoms of pyramidal or extrapyramidal motor dysfunction, behavioral and psychiatric abnormalities, cognitive impairment, and speech difficulty. Brain imaging abnormalities were common, and initial diagnosis category was significantly associated with white matter hyperintensities, white matter calcifications, and ventricular enlargement.

**Discussion:**

In this literature analysis, ALSP was frequently misdiagnosed. Improving awareness of this condition and distinguishing it from other conditions with overlapping presenting symptoms is important for timely management of a rapidly progressive disease such as ALSP.

## 1 Introduction

Adult-onset leukoencephalopathy with axonal spheroids and pigmented glia (ALSP) is a rare, hereditary, autosomal dominant neurodegenerative disorder with typical onset between 40 and 50 years of age (mean age, 43 years [range: 18–78 years]) (1, 2). Loss-of-function mutations in the colony-stimulating factor-1 receptor gene (*CSF1R*) cause the morphologic abnormalities that are characteristic of ALSP, including distended neuronal axons, pigmented glial cells, and demyelination of cerebral white matter (3, 4). The treatment of ALSP remains an unmet medical need, as no symptomatic or disease-modifying therapies are currently approved to reverse, delay, or stop the progression of this disabling disorder (3, 4). The natural course of ALSP is marked by rapidly progressive and debilitating cognitive impairment, moderate to severe motor dysfunction, and neuropsychiatric complications, leading to impaired quality of life and death within approximately 6–8 years from symptom onset (1, 4, 5).

Symptom onset and progression among patients with ALSP is variable, even within the same family, and differential diagnosis of ALSP can be challenging due to the overlapping presentation of symptoms and radiologic features that can mimic other neurodegenerative and white matter diseases (inflammatory, vascular, or genetic) (4–6). A previously conducted comprehensive review of the literature examined disorders with clinical symptoms that overlap with ALSP (3), highlighting that the differential diagnosis of ALSP should include Alzheimer’s disease, cerebral autosomal dominant arteriopathy with subcortical infarcts and leukoencephalopathy (CADASIL), atypical Parkinson’s disease, other leukodystrophies, frontotemporal dementia, progressive multiple sclerosis, and vascular dementia (3–5).

Validated diagnostic criteria for ALSP that were developed through a retrospective case study combine specific core features, exclusionary findings, and supportive findings to generate the diagnosis of ALSP as definite, probable, or possible (7); however, independent analyses have suggested that these criteria demonstrate poor specificity (8, 9), which may be because they were designed to avoid missing atypical ALSP cases (8). Genetic testing for confirmation of a *CSF1R* mutation is required for a definitive diagnosis, but limited accessibility to genetic testing may delay diagnosis. Another diagnostic model for ALSP has been evaluated, but its applicability is limited by a restrictive neuroimaging and neuropathologic approach (10).

Because no regulatory-approved disease-modifying therapies are currently available for ALSP (3), rapid and accurate diagnosis of ALSP is critical for initiating clinical care as well as for the design of future studies to test experimental and interventional therapies. Thus, the objective of this retrospective literature analysis was to leverage a comprehensive review of published cases from the peer-reviewed literature (3) to examine the frequency of misdiagnosis among a cohort of patients with ALSP. Discussion of therapies that are commonly used to temporarily relieve the motor, mood, and behavior symptoms of ALSP as well as experimental approaches, such as hematopoietic stem cell transplantation and others, are outside the scope of this current work but are covered in detail elsewhere in the literature (3, 4, 11).

## 2 Methods

A cohort of patients with ALSP was collated from individual case reports and series identified by a literature search. Limited findings from this analysis have been reported elsewhere (3), but this is the first report of the full methodology.

### 2.1 Search strategy

ALSP case series and case reports were identified via a PubMed search using the following search terms: “adult-onset leukodystrophy with neuroaxonal spheroids and pigmented glia,” “adult-onset leukoencephalopathy with axonal spheroids and pigmented glia,” “ALSP,” “hereditary diffuse leukoencephalopathy with spheroids,” “HDLS,” “pigmentary orthochromatic leukodystrophy,” “POLD,” and “CSF1R-related leukoencephalopathy.” Search results were limited to English-language articles published between January 1, 1980, and March 22, 2022. Abstracts of the identified articles were manually curated for eligible publications that clearly delineated clinical details in case reports and case series, after which the remaining publications were reviewed in detail by 4 reviewers (employees of and consultants to Vigil Neuroscience, Inc.) to confirm eligibility.

### 2.2 Eligibility criteria for case reports and series

Articles eligible for inclusion in this analysis were case series and case reports with clearly delineated clinical details of adults aged ≥ 18 years (living or deceased) with a diagnosis of ALSP, pigmentary orthochromatic leukodystrophy, or hereditary diffuse leukoencephalopathy with spheroids (HDLS) confirmed by genetic testing for the *CSF1R* mutation, brain imaging, and/or brain histopathology. Publications that did not have adequate clinical data, included data from non-case studies, or that confirmed the presence of mutation in the alanyl-tRNA synthetase gene (*AARS*) via genetic testing were excluded. Among the excluded cases were members from the Swedish family in which HDLS was originally identified, since genetic testing has recently shown the likely etiological cause in affected members of this family to be a genetic variant of *AARS*, rather than *CSF1R* (12).

### 2.3 Data extraction

Individual patient data were extracted from a cohort of 291 patients with ALSP from 93 published case reports and series. The reviewers entered the data into a master spreadsheet under the appropriate demographic and clinical characteristic headings. Prior to statistical analysis, all extracted data were independently reviewed and examined for accuracy. Disputes regarding data accuracy were resolved through consultation and consensus among reviewers. Data from the same patient in multiple case reports were extracted and entered only once into the master spreadsheet to avoid duplication.

### 2.4 Statistical analysis

Categorical patient information collected from each case report included the following: demographics (sex, family history, geographic region), *CSF1R* mutation and its corresponding exon position (intron, within exons 18–21, outside of exons 18–21) and protein region (signal peptide, immunoglobulin-like domain, juxtamembrane domain, tyrosine kinase domain 1, kinase insertion domain, tyrosine kinase domain 2, carboxyl-terminal domain), initial diagnosis category (ALSP, frontotemporal dementia, multiple sclerosis, cardiovascular disease familial leukoencephalopathy, adult-onset leukodystrophy, Alzheimer’s disease, nonspecific neurodegeneration or dementia; Supplementary Table 1), clinical symptoms (cognitive impairment, pyramidal motor abnormalities, extrapyramidal motor abnormalities, behavioral and psychiatric dysfunction, speech dysfunction) at presentation and during progression, abnormal brain imaging (atrophy, corpus callosum abnormalities, ventricular enlargement, white matter hyperintensities, white matter calcification), concurrent medications, and clinical outcome assessment scores. Ages of onset and death, disease duration if alive, and survival time (years) were collected as numerical (continuous) variables. Concurrent medications and clinical outcome assessments were not analyzed due to a paucity of data.

Analyses were descriptive for continuous data (mean, standard deviation [SD], median, range) and categorical data (frequency, percentage). Categorical data for demographics, symptoms, and brain imaging were also stratified by initial diagnosis category. An analysis of variance model was used to evaluate whether initial diagnosis category varied based on the frequency distribution between categorical variables for patient demographics, exon position of *CSF1R* mutation, protein region of *CSF1R* mutation, symptoms, or brain imaging. Fisher’s exact test or Chi-square testing was used to test for statistical significance at an α level of 0.05 and marginal significance at a level of 0.10.

## 3 Results

Data for 291 patients with a confirmed diagnosis of ALSP were extracted from 93 eligible published case reports (1, 2, 11, 13–102). Of these, only 14 cases from 7 articles (39, 58, 64, 79, 85, 86, 94) were characterized prior to the identification of *CSF1R* mutation as the genetic basis of ALSP (published in 2011) (72).

Overall, the mean (SD) age of ALSP onset was 43.2 (11.6) years, cases were approximately evenly divided between females (48.1%) and males (42.6%), and a family history of ALSP was reported for more than half (59.1%) (Table 1). Approximately one-third of cases in this analysis were from Asia (36.1%), Europe (32.6%), and North America (24.4%). *CSF1R* mutations were most often located within exons 18–21 (55.7%) compared with elsewhere (outside of exons 18–21, 24.1%; intronic, 7.9%) and most mapped to either tyrosine kinase domain 2 (67.7%) or domain 1 (15.1%) of the CSF1R protein. The most frequently reported initial symptoms were cognitive impairment (47.1%) and behavioral and psychiatric abnormalities (26.8%), followed by extrapyramidal motor symptoms (16.2%), pyramidal motor dysfunction (11.7%), and speech difficulty (11.3%).

**Table 1:** Demographics and initial symptoms of patients with ALSP.

Variables^a^	All patients (*N* = 291)
**Age of onset**	
Mean (SD)	43.2 (11.6)
Median (min, max)	42.0 (18.0, 86.0)
≤42 years	143 (49.1)
>42 years	136 (46.7)
Missing	12 (4.1)
**Sex**	
Female	140 (48.1)
Male	124 (42.6)
Missing	27 (9.3)
**Family history**	
Yes	172 (59.1)
No	76 (26.1)
Missing	43 (14.8)
**Geographic region**	
Asia	105 (36.1)
Europe	95 (32.6)
North America	71 (24.4)
Missing	20 (6.9)
**Mode of inheritance**	
Autosomal dominant	103 (35.4)
*De novo*	62 (21.3)
Maternal	2 (0.7)
Unknown/missing	124 (42.6)
**Exon position of mutation**	
Exon (within 18–21)	162 (55.7)
Exon (outside 18–21)	70 (24.1)
Intron	23 (7.9)
Missing	36 (12.4)
**Kinase domain mutation**	
Yes	241 (82.8)
No	13 (4.5)
Missing	37 (12.7)
**Protein region of mutation**	
Signal peptide	2 (0.7)
Ig-like domains	3 (1.0)
Juxtamembrane domain	3 (1.0)
Tyrosine kinase domain 1	44 (15.1)
Kinase insertion domain	2 (0.7)
Tyrosine kinase domain 2	197 (67.7)
C-terminal domain	3 (1.0)
Missing	37 (12.7)
**Initial symptoms** ^b^	
Cognitive impairment	137 (47.1)
Behavioral and psychiatric	78 (26.8)
Extrapyramidal motor	47 (16.2)
Pyramidal motor	34 (11.7)
Speech difficulty	33 (11.3)
Missing	69 (23.7)

ALSP, adult-onset leukoencephalopathy with axonal spheroids and pigmented glia; Ig, immunoglobulin; SD, standard deviation.

a Data are expressed as n (%) unless otherwise indicated.

b Initial symptom categories do not add up to 100% because categories are not mutually exclusive.

**Figure 1:**
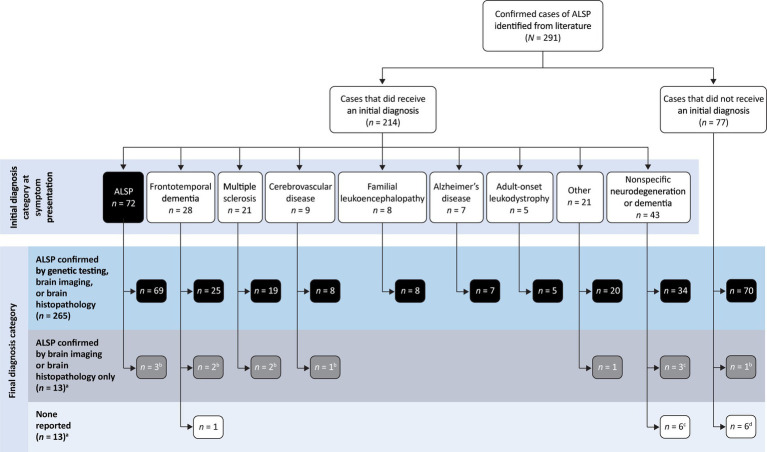
Summary of initial and final diagnosis categories. ^a^14 cases from 7 publications were published prior to the identification of *CSF1R* mutation as the genetic basis of ALSP. ^b^All, ^c^2, or ^d^1 cases published prior to the identification of *CSF1R* mutation as the genetic basis of ALSP.

Table 2 shows the distribution of initial diagnoses within this cohort. Within the cohort of 291 patients, 77 cases (26.5%) did not report an initial diagnosis and only 72 cases (24.7%) received an accurate initial diagnosis of ALSP. Initial misdiagnosis with several other neurodegenerative and white matter diseases was common, including frontotemporal dementia (9.6%), multiple sclerosis (7.2%), cerebrovascular disease (3.1%), familial leukoencephalopathy (2.7%), Alzheimer’s disease (2.4%), adult-onset leukodystrophy (1.7%), and other miscellaneous disorders (7.2%). In addition, 14.8% of cases were diagnosed nonspecifically with multiple disorders or using broader phenotypic terms (e.g., “leukodystrophy,” “leukoencephalopathy,” or “dementia”). Of the 219 cases (75.3%) that were initially mis- or undiagnosed (Figure 1), 206 cases (94.1%) were later confirmed as ALSP by immunohistology, imaging, and/or genetic testing, whereas a final diagnosis was not reported for 13 cases (5.9%). Of the 206 ALSP-confirmed cases, only 13 cases were confirmed without genetic testing, 11 of which were reported prior to the introduction of the genetic test.

**Table 2:** Initial diagnosis categories of patients.

Initial diagnosis category^a^	Patients, n (%) (*N* = 291)	Age of onset, years Mean (min, max)
ALSP	72 (24.7)	44.1 (18.0, 86.0)
Frontotemporal dementia	28 (9.6)	50.2 (33.0, 71.0)^d^
Multiple sclerosis	21 (7.2)	33.5 (20.0, 47.0)
Cerebrovascular disease	9 (3.1)	38.2 (21.0, 57.0)
Familial leukoencephalopathy	8 (2.7)	48.6 (40.0, 60.0)
Alzheimer’s disease	7 (2.4)	54.0 (40.0, 78.0)
Adult-onset leukodystrophy	5 (1.7)	36.8 (25.0, 45.0)
Nonspecific neurodegeneration or dementia^b^	43 (14.8)	42.2 (18.0, 63.0)
Other^c^	21 (7.2)	45.7 (22.0, 67.0)^d^
Missing	77 (26.5)	41.6 (22.9, 70.0)^e^

ALSP, adult-onset leukoencephalopathy with axonal spheroids and pigmented glia; CADASIL, cerebral autosomal dominant arteriopathy with subcortical infarcts and leukoencephalopathy; CNS, central nervous system.

a Diagnosis category reflects verbatim terminology from case reports (Supplementary Table 1).

b Includes nonspecific (“leukodystrophy,” “leukoencephalopathy,” “dementia”) or multiple initial diagnoses.

c Includes corticobasal syndrome (*n* = 7), CADASIL (*n* = 5), parkinsonism (*n* = 2), and Binswanger disease, cervical spondylotic myelopathy, CNS lesions related to celiac disease, lumbosacral spondylolisthesis, neuropsychiatric systemic lupus erythematosus, pulmonary tuberculosis, and spasticity (each *n* = 1).

d Age missing for 1 patient.

e Age missing for 10 patients.

Analysis of the influence of initial diagnosis category on demographic and disease characteristics is shown in Table 3. Initial diagnosis categories varied significantly based on age (< 42 vs. ≥ 42; *p* < 0.001), with initial diagnoses categorized as Alzheimer’s disease and frontotemporal dementia tending toward older ages (mean age ≥ 50 years) and as multiple sclerosis tending toward younger ages (mean age < 40 years). The initial diagnosis category also significantly differed based on having a family history of ALSP (*p* = 0.046); higher proportions of patients with a family history of ALSP received an initial diagnosis within the categories of familial leukoencephalopathy (87.5%) and cerebrovascular disease (77.8%) compared with multiple sclerosis (47.6%) and adult-onset leukoencephalopathy (20.0%). Initial diagnosis category was also significantly associated with geographical region (*p* < 0.001), mode of inheritance (*p* < 0.001), and presenting symptoms of pyramidal (*p* = 0.027) or extrapyramidal (*p* = 0.020) motor dysfunction, behavioral and psychiatric abnormalities (*p* = 0.047), cognitive impairment (*p* = 0.007), and speech difficulty (*p* = 0.003). Among the 34 cases with initial diagnoses categorized as Alzheimer’s disease or frontotemporal dementia, none were reported to have pyramidal motor symptoms and only 1 case had extrapyramidal motor dysfunction, compared with higher proportions of patients with motor symptoms (9.5–30.2%) across the other initial diagnosis categories. Behavioral and psychiatric abnormalities and cognitive impairment were each observed in only 1 patient with an initial diagnosis in the cerebrovascular disease category compared with higher proportions of patients across the other initial diagnosis categories (behavioral and psychiatric abnormalities, 12.5–52.4%; cognitive impairment, 41.9–87.5%). Initial diagnosis did not vary based on sex or location of *CSF1R* mutation (exon number, kinase domain, or protein region).

**Table 3:** Demographics analyzed by initial diagnosis category.

Variables^a^	ALSP (*n* = 72)	Frontotemporal dementia (*n* = 27)^b^	Multiple sclerosis (*n* = 21)	Cerebrovascular disease (*n* = 9)	Familial leukoencephalopathy (*n* = 8)	Alzheimer’s disease (*n* = 7)	Adult-onset leukodystrophy (*n* = 5)	Nonspecific neurodegeneration or dementia (*n* = 43)	Other (*n* = 20)^b^	Missing (*n* = 67)^b^	*p* value
**Age of onset**											
Mean (SD)	44.1 (12.7)	50.3 (8.5)	33.4 (6.7)	38.2 (11.5)	48.6 (6.6)	54.0 (15.3)	36.8 (9.1)	42.2 (10.6)	45.7 (12.6)	41.5 (10.3)	<0.001
Median (min, max)	42.5 (18.0, 86.0)	50.0 (33.0, 71.0)	36.0 (20.0, 47.0)	40.0 (21.0, 57.0)	49.0 (40.0, 60.0)	52.0 (40.0, 78.0)	42.0 (25.0, 45.0)	43.0 (18.0, 63.0)	43.8 (22.0, 67.0)	40 (22.9, 70.0)	
≤42 years	36 (50.0)	5 (18.5)	20 (95.2)	5 (55.6)	2 (25.0)	3 (42.9)	3 (60.0)	20 (46.5)	10 (50.0)	39 (58.2)	<0.001
>42 years	36 (50.0)	22 (81.5)	1 (4.8)	4 (44.4)	6 (75.0)	4 (57.1)	2 (40.0)	23 (53.5)	10 (50.0)	28 (41.8)	
**Sex**											
Female	36 (50.0)	10 (37.0)	16 (76.2)	6 (66.7)	3 (37.5)	3 (42.9)	2 (40.0)	21 (48.8)	8 (40.0)	32 (47.8)	0.09
Male	36 (50.0)	14 (51.9)	2 (9.5)	3 (33.3)	5 (62.5)	3 (42.9)	1 (20.0)	16 (37.2)	9 (45.0)	35 (52.2)	
Missing	0	3 (11.1)	3 (14.3)	0	0	1 (14.3)	2 (40.0)	6 (14.0)	3 (15.0)	0	
**Family history**											
Yes	39 (54.2)	20 (74.1)	10 (47.6)	7 (77.8)	7 (87.5)	4 (57.1)	1 (20.0)	27 (62.8)	14 (70.0)	40 (59.7)	0.046
No	29 (40.3)	4 (14.8)	8 (38.1)	2 (22.2)	1 (12.5)	2 (28.6)	4 (80.0)	8 (18.6)	2 (10.0)	16 (23.9)	
Missing	4 (5.6)	3 (11.1)	3 (14.3)	0	0	1 (14.3)	0	8 (18.6)	4 (20.0)	11 (16.4)	
**Geographical region**											
Asia	38 (52.8)	4 (14.8)	5 (23.8)	3 (33.3)	5 (62.5)	4 (57.1)	0	5 (11.6)	4 (20.0)	36 (53.7)	<0.001
Europe	9 (12.5)	14 (51.9)	9 (42.9)	1 (11.1)	3 (37.5)	0	5 (100)	18 (41.9)	8 (40.0)	27 (40.3)	
North America	25 (34.7)	9 (33.3)	7 (33.3)	5 (55.6)	0	3 (42.9)	0	20 (46.5)	8 (40.0)	4 (6.0)	
**Mode of inheritance**											
Autosomal dominant	32 (44.4)	13 (48.1)	8 (38.1)	6 (66.7)	5 (62.5)	4 (57.1)	1 (20.0)	14 (32.6)	9 (45.0)	9 (13.4)	<0.001
*De novo*	34 (47.2)	2 (7.4)	6 (28.6)	1 (11.1)	0	2 (28.6)	4 (80.0)	7 (16.3)	1 (5.0)	5 (7.5)	
Maternal	0	0	0	0	0	0	0	0	0	2 (3.0)	
Unknown/missing	6 (8.3)	12 (44.4)	7 (33.3)	2 (22.2)	3 (37.5)	1 (14.3)	0	22 (51.2)	10 (50.0)	51 (76.1)	
**Exon number mutations**											
Exon (within 18–21)	38 (52.8)	16 (59.3)	10 (47.6)	5 (55.6)	6 (75.0)	3 (42.9)	1 (20.0)	25 (58.1)	11 (55.0)	42 (62.7)	0.246
Exon (outside 18–21)	24 (33.3)	4 (14.8)	4 (19.0)	1 (11.1)	2 (25.0)	1 (14.3)	3 (60.0)	7 (16.3)	3 (15.0)	15 (22.4)	
Intron	6 (8.3)	4 (14.8)	4 (19.0)	1 (11.1)	0	1 (14.3)	1 (20.0)	1 (2.3)	2 (10.0)	3 (4.5)	
Missing	4 (5.6)	3 (11.1)	3 (14.3)	2 (22.2)	0	2 (28.6)	0	10 (23.3)	4 (20.0)	7 (10.4)	
**Kinase domain mutation**											
Yes	60 (83.3)	24 (88.9)	17 (81.0)	7 (77.8)	8 (100)	4 (57.1)	5 (100)	31 (72.1)	16 (80.0)	60 (89.6)	0.056
No	8 (11.1)	0	1 (4.8)	0	0	1 (14.3)	0	1 (2.3)	0	0	
Missing	4 (5.6)	3 (11.1)	3 (14.3)	2 (22.2)	0	2 (28.6)	0	11 (25.6)	4 (20.0)	7 (10.4)	
**Protein region mutations**											
Signal peptide	1 (1.4)	0	0	0	0	0	0	1 (2.3)	0	0	0.441
Ig-like domains	3 (4.2)	0	0	0	0	0	0	0	0	0	
Juxtamembrane domain	1 (1.4)	0	1 (4.8)	0	0	0	0	0	0	0	
Tyrosine kinase domain 1	12 (16.7)	3 (11.1)	6 (28.6)	2 (22.2)	1 (12.5)	0	2 (40.0)	2 (4.7)	3 (15.0)	9 (13.4)	
Kinase insertion domain	1 (1.4)	0	0	0	0	1 (14.3)	0	0	0	0	
Tyrosine kinase domain 2	48 (66.7)	21 (77.8)	11 (52.4)	5 (55.6)	7 (87.5)	4 (57.1)	3 (60.0)	29 (67.4)	13 (65.0)	51 (76.1)	
C-terminal domain	2 (2.8)	0	0	0	0	0	0	0	0	0	
Missing	4 (5.6)	3 (11.1)	3 (14.3)	2 (22.2)	0	2 (28.6)	0	11 (25.6)	4 (20.0)	7 (10.4)	
**Initial symptoms** ^c^											
Cognitive impairment	49 (68.1)	16 (59.3)	11 (52.4)	1 (11.1)	7 (87.5)	6 (85.7)	4 (80.0)	18 (41.9)	11 (55.0)	13 (19.4)	0.007
Behavioral and psychiatric	26 (36.1)	8 (29.6)	11 (52.4)	1 (11.1)	1 (12.5)	3 (42.9)	2 (40.0)	18 (41.9)	3 (15.0)	5 (7.5)	0.047
Extrapyramidal motor	11 (15.3)	1 (3.7)	2 (9.5)	2 (22.2)	1 (12.5)	0	2 (40.0)	13 (30.2)	4 (20.0)	11 (16.4)	0.020
Pyramidal motor	7 (9.7)	0	6 (28.6)	2 (22.2)	0	0	0	8 (18.6)	5 (25.0)	6 (9.0)	0.027
Speech difficulty	5 (6.9)	1 (3.7)	2 (9.5)	2 (22.2)	0	1 (14.3)	0	6 (14.0)	3 (15.0)	13 (19.4)	0.003
Missing	0	8 (29.6)	2 (9.5)	1 (11.1)	0	0	0	6 (14.0)	3 (15.0)	40 (59.7)	N/A

ALSP, adult-onset leukoencephalopathy with axonal spheroids and pigmented glia; Ig, immunoglobulin; SD, standard deviation.

a Data are expressed as n (%) unless otherwise indicated.

b Cases with missing age (*n* = 12) were not included in the analysis of variance.

c Initial symptom categories do not add up to 100% because categories are not mutually exclusive.

Table 4 presents brain imaging abnormalities of patients stratified by all initial diagnosis categories. Initial diagnosis category varied significantly based on white matter hyperintensities, white matter calcification, and ventricular enlargement. No brain imaging abnormalities were reported for any cases with an initial diagnosis category of familial leukoencephalopathy, and those initially diagnosed with ALSP had the highest (corpus callosum irregularities, 44.4% vs. 0–40.0%) or second highest (white matter hyperintensities, 91.7% vs. 0–100%; white matter calcification, 38.9% vs. 0–44.4%; ventricular enlargement, 27.8% vs. 0–28.6%) proportions of brain imaging irregularities compared with the other initial diagnosis categories.

**Table 4:** Brain imaging abnormalities analyzed by initial diagnosis category.

Variables, n (%)	ALSP (*n* = 72)	Frontotemporal dementia (*n* = 27)^a^	Multiple sclerosis (*n* = 21)	Cerebrovascular disease (*n* = 9)	Familial leukoencephalopathy (*n* = 8)	Alzheimer’s disease (*n* = 7)	Adult-onset leukodystrophy (*n* = 5)	Nonspecific neurodegeneration or dementia (*n* = 43)	Other (*n* = 20)^a^	Missing (*n* = 67)^a^	*p* value
White matter hyperintensities	66 (91.7)	14 (51.9)	17 (81.0)	5 (55.6)	0	3 (42.9)	5 (100)	25 (58.1)	13 (65.0)	54 (80.6)	<0.001
Corpus callosum irregularities^b^	32 (44.4)	5 (18.5)	8 (38.1)	3 (33.3)	0	2 (28.6)	2 (40.0)	6 (14.0)	2 (10.0)	36 (53.7)	0.053
Atrophy	27 (37.5)	10 (37.0)	7 (33.3)	3 (33.3)	0	2 (28.6)	3 (60.0)	12 (27.9)	4 (20.0)	26 (38.8)	0.516
White matter calcification	28 (38.9)	1 (3.7)	3 (14.3)	4 (44.4)	0	0	0	5 (11.6)	2 (10.0)	3 (4.5)	<0.001
Ventricular enlargement	20 (27.8)	3 (11.1)	1 (4.8)	1 (11.1)	0	2 (28.6)	0	3 (7.0)	2 (10.0)	8 (11.9)	<0.001

ALSP, adult-onset leukoencephalopathy with axonal spheroids and pigmented glia.

a Cases with missing age (*n* = 12) were not included in the analysis of variance.

b Thinning and/or hyperintensities.

## 4 Discussion

To our knowledge, the findings of this analysis of published cases represent the largest case series to date of patients with ALSP. Initial misdiagnosis of ALSP was common, with an accurate initial diagnosis achieved in only 24.7% of patients with ALSP. This is likely due in part to symptoms that overlap with other disorders, including early-onset Alzheimer’s disease (executive functioning, memory, language, and personality changes), frontotemporal dementia (problems with social behavior, personality, and language), familial leukoencephalopathy (behavioral or cognitive decline), multiple sclerosis (cognitive problems, language problems, or motor neuron impairment), and cerebrovascular diseases (speech difficulty, confusion, and memory derangement) (5, 103–107).

A rapid, accurate diagnosis of ALSP is critical in providing supportive symptom management and potentially allowing for early therapeutic intervention. Definitive diagnosis of ALSP can be confirmed with genetic testing for pathogenic *CSF1R* mutations in the clinical context of characteristic symptoms (cognitive impairment, moderate to severe motor dysfunction, and neuropsychiatric symptoms), typical brain imaging abnormalities, and in some cases with a supportive family history (as a subset of patients have *de novo* patterns of inheritance) (3, 7). Although genetic testing is crucial for a definitive diagnosis of ALSP, use in clinical practice may be limited due to cost and availability (7).

Examination of categorical data for patients stratified by initial diagnosis category suggested that age, family history, geographic region, and mode of inheritance were associated with the selection of initial diagnosis. Similarly, initial diagnoses appear to have been influenced by presenting symptoms such as pyramidal or extrapyramidal motor dysfunction, behavioral and psychiatric abnormalities, cognitive impairment, and speech difficulty. Stratification of brain imaging abnormalities by initial diagnosis category also demonstrated statistically significant associations between initial diagnosis category and the presence of white matter hyperintensities, ventricular enlargement, white matter calcification, and corpus callosum irregularities. Abnormal diffusion on brain MRI, which can persist for extended periods of time, has also been observed in patients with ALSP in recent literature (1, 7, 11, 20), but the presence of diffusion abnormalities was not systematically examined in this analysis.

These data are consistent with current evidence indicating the importance of brain imaging in accurately diagnosing ALSP (2–4). The diagnostic criteria developed for ALSP combine brain imaging with presenting characteristics such as patient age, symptoms (cognitive, psychiatric, and motor dysfunction), and inheritance patterns (autosomal dominant or sporadic) (7). Retrospective application of these criteria to more than 150 patients with ALSP and *CSF1R*-positive mutations, *CSF1R*-negative leukoencephalopathies, or CADASIL showed a sensitivity of 99% to accurately detect probable or possible cases and an adequate specificity of 88% to exclude non-ALSP cases (7).

This retrospective literature analysis is subject to several limitations. The development of this cohort and associated individual patient data was based entirely upon published case reports and case series, which are contingent upon the reporting standards associated with original patient medical records; thus, potential errors or omissions in the clinical descriptions may have resulted in inaccurate, incomplete, or missing clinical assessments of some symptoms of ALSP. Further, the geographic locations of the authors and clinics providing each case report exhibited considerable variation, which may have generated heterogeneity in interpretation and reporting of clinical manifestations.

For this cohort, the initial diagnosis information extracted from the primary sources constituted an unavoidably heterogeneous dataset, with some imprecision in reporting in the published literature, that necessitated application of discrete categories for interpretation; this analysis is limited by the subjective nature and potential for overlap inherent in such categorization. This analysis also reflects the state of the ALSP literature over this time period. Initial diagnosis data were missing in 26.5% of cases, and the available data for another 14.8% of cases necessitated a judgment be made as to whether nonspecific or multiple initial diagnoses (i.e., “leukodystrophy” or “Alzheimer’s disease, atypical CADASIL, or atypical multiple sclerosis”) should be categorized as true misdiagnoses or as broad tentative phenotypic diagnoses that should be considered a correct initial diagnosis that was given with the intent for later revision. Furthermore, a handful of cases were reported prior to the introduction of genetic testing for a *CSF1R* mutation that is now necessary to confirm an ALSP diagnosis, which may also have added an additional, albeit small, element of uncertainty.

An analysis of the literature often includes measures to assess the risk of reporting bias arising from study design, conduct, or analyses; however, the individual patient-level data that comprise this dataset are derived from case series and case reports. Therefore, assessments of bias in study design, conduct, or analyses are not applicable to this cohort. Because the sampling procedure for this cohort was limited to cases published in peer-reviewed journals, these results are inherently subject to publication bias. Not all clinical cases are necessarily or systematically published in the peer-reviewed literature, and published case reports may sometimes tend to highlight unique or “interesting” clinical features. For these reasons, a cohort based entirely of published case reports may not represent the clinical experience of all patients living with ALSP, and the estimates obtained from this cohort may not be fully generalizable to all patients living with this disease. Absolute rates of ALSP misdiagnosis could differ from that reported in this analysis, and future comparisons of this cohort to new patient cases and/or clinical trial data may be necessary for broader generalizability.

In conclusion, despite the application of diagnostic criteria to distinguish ALSP from other disorders that present similarly and the development of a genetic test for the *CSF1R* mutation, diagnosis of ALSP remains challenging. Previous studies have estimated a prevalence of at least 10,000 cases of ALSP in the United States (61, 108, 109); however, only a small number of patients with confirmed ALSP have been currently identified (3, 109), which may be due, at least in part, to the high rate of initial misdiagnosis. Therefore, increased awareness of ALSP and further investigation and characterization into its presenting symptoms are needed to improve diagnostic accuracy of this debilitating disorder.

## Trial registration

This review of published ALSP case reports was registered in the Research Registry Database under unique identification number 1251.

## Data availability statement

The raw data supporting the conclusions of this article will be made available by the authors, without undue reservation.

## Ethics statement

This study was exempt from requiring ethics approval or written informed consent because it is a retrospective analysis of previously collected and published data.

## Author contributions

SP: Conceptualization, Methodology, Data curation, Writing – review & editing, Investigation. JG: Writing – review & editing, Investigation. TK: Writing – review & editing, Investigation. TI: Writing – review & editing, Investigation. AP: Writing – review & editing, Data curation. AM: Writing – review & editing, Data curation, Investigation. FF: Formal analysis, Writing – review & editing, Investigation. ZW: Writing – review & editing, Investigation, Data curation.
