# Supplementary material for: Clinical presentation and diagnosis of adult-onset leukoencephalopathy with axonal spheroids and pigmented glia: a literature analysis of case studies
Source: Front Neurol. 2024 Mar 11;15:1320663. doi: 10.3389/fneur.2024.1320663 (PMC10962389; doi:10.3389/fneur.2024.1320663)
Supplement: Supplementary file 1 [file Table_1.DOCX]

**Supplemental Material**

**SUPPLEMENTAL TABLE 1. Categorization of verbatim terms used for initial diagnosis categories.**

| **Initial Diagnosis Category** | **Verbatim Term(s) Extracted from Primary Sources** |
| --- | --- |
| Adult-onset leukodystrophy (*n* = 5) | Adult onset leukodystrophy (*n* = 4)  Adult onset leukodystrophy of unknown cause (*n* = 1) |
| ALSP (*n* = 72) | ALSP (*n* = 38)  HDLS (*n* = 29)  Hereditary diffuse leukoencephalopathy with spheroids (*n* = 5) |
| Alzheimer’s disease (*n* = 7) | Alzheimer's disease (*n* = 6)  Juvenile Alzheimer's disease (*n* = 1) |
| Cerebrovascular disease (*n* = 9) | Acute ischemic stroke (*n* = 1)  Epilepsy or cerebrovascular disorder (*n* = 1)  Hydrocephalus (*n* = 1)  Inherited microangiopathy (*n* = 1)  Microvascular leukoencephalopathy (*n* = 1)  Multiple cerebral infarction and hydrocephalus (*n* = 1)  Possible stroke disorder (*n* = 1)  Psychiatric disease or normal pressure hydrocephalus (*n* = 1)  Stroke (*n* = 1) |
| Familial leukoencephalopathy (*n* = 8) | Familial leukoencephalopathy (*n* = 5)  Vascular leukoencephalopathy (inherited) (*n* = 3) |
| Frontotemporal dementia (*n* = 28) | Behavioral variant of frontotemporal dementia (*n* = 1)  Frontal lobe dementia (*n* = 1)  Frontotemporal degeneration (*n* = 1)  Frontotemporal dementia (*n* = 12)  Frontotemporal dementia with motor neuron disease (*n* = 1)  FTD (*n* = 8)  FTLD (genetic form) (*n* = 3)  Primary progressive aphasia (*n* = 1) |
| Missing (*n* = 77) | [No terms reported] (*n* = 77) |
| Multiple sclerosis (*n* = 21) | Atypical multiple sclerosis (*n* = 2)  Multiple sclerosis (*n* = 15)  Possible MS (*n* = 1)  PPMS (*n* = 1)  Primary progressive multiple sclerosis (*n* = 1)  Progressive terminal MS (*n* = 1) |
| Nonspecific neurodegeneration or dementia (*n* = 43) | Neuroaxonal leukodystrophy based on family history (*n* = 1)  Alzheimer disease, Pick disease, multi-infarct dementia (*n* = 1)  Alzheimer or other frontotemporal dementia (*n* = 1)  Alzheimer's disease, atypical CADASIL, atypical multiple sclerosis (*n* = 1)  Alzheimer's disease, CADASIL (*n* = 1)  Atypical dementia (*n* = 1)  CADASIL, CARASIL, AD (*n* = 1)  Dementia with Lewy bodies and parkinsonism (*n* = 1)  Demyelinating disorder (*n* = 1)  Early-onset dementia (*n* = 1)  Frontotemporal dementia of atypical CADASIL (*n* = 2)  Frontotemporal dementia or cerebrovascular disease (*n* = 1)  Frontotemporal dementia, Alzheimer's disease (*n* = 1)  Frontotemporal dementia, Alzheimer's disease, CADASIL (*n* = 1)  Inflammatory demyelination (*n* = 1)  Leukodystrophy (*n* = 6)  Leukodystrophy of unclear cause (*n* = 1)  Leukodystrophy of unknown origin (*n* = 3)  Leukodystrophy perhaps metachromatic (*n* = 1)  Leukoencephalopathy with calcifications and cysts (*n* = 2)  Leukoencephalopathy with unknown etiology (*n* = 1)  Multiple sclerosis or atypical Parkinson's disease (*n* = 1)  Multiple sclerosis or vascular leukoencephalopathy (*n* = 1)  Neuromyelitis optica, atypical Parkinson's disease, atypical multiple sclerosis, progressive supranuclear palsy (*n* = 1)  Orthochromatic leukodystrophy (*n* = 2)  Pallido-ponto-nigral degeneration (*n* = 1)  Parkinson's disease, progressive supranuclear palsy,  multiple sclerosis (*n* = 1)  Presenile dementia (*n* = 1)  Spinocerebellar ataxia, dentatorubral-pallidoluysian atrophy,  Alexander disease, Niemann-Pick disease (*n* = 1)  Undetermined leukoencephalopathy (*n* = 2)  Vascular dementia (*n* = 1)  Vasculitis, mitochondrial encephalopathy or leukodystrophy (*n* = 1) |
| Other (*n* = 21) | Atypical CADASIL (*n* = 1)  Binswanger disease (*n* = 1)  CADASIL (*n* = 4)  CBDS (*n* = 1)  Cervical spondylotic myelopathy (*n* = 1)  CNS lesions related to celiac disease (*n* = 1)  Corticobasal degeneration (*n* = 1)  Corticobasal syndrome (*n* = 5)  Lumbosacral spondylolisthesis (*n* = 1)  NP-SLE (neuropsychiatric lupus) (*n* = 1)  Parkinsonism (*n* = 2)  Pulmonary tuberculosis (*n* = 1)  Spasticity (*n* = 1) |

AD, Alzheimer’s disease; ALSP, adult-onset leukoencephalopathy with axonal spheroids and pigmented glia; CADASIL, cerebral autosomal dominant arteriopathy with subcortical infarcts and leukoencephalopathy; CARASIL, cerebral autosomal recessive arteriopathy with subcortical infarcts and leukoencephalopathy; CBDS, corticobasal degeneration syndrome; CNS, central nervous system; FTD, frontotemporal dementia; FTLD, frontotemporal lobar degeneration; HDLS, hereditary diffuse leukoencephalopathy with spheroids; MS, multiple sclerosis; NP-SLE, neuropsychiatric systemic lupus erythematosus; PPMS, primary progressive multiple sclerosis.
